# Patients’ and clinicians’ experiences with stratified exercise therapy in knee osteoarthritis: a qualitative study

**DOI:** 10.1186/s12891-022-05496-2

**Published:** 2022-06-09

**Authors:** J. Knoop, J. W. de Joode, H. Brandt, J. Dekker, R. W. J. G. Ostelo

**Affiliations:** 1 Vrije Universiteit Amsterdam, Department of Health Sciences, Amsterdam, De Boelelaan 1105, Amsterdam, 1081 HV Netherlands; 2 Amsterdam UMC, Location VUmc, Department of Rehabilitation Medicine, Amsterdam, Netherlands; 3 Vrije Universiteit Amsterdam, Amsterdam Movement Sciences, Amsterdam, Netherlands; 4 Amsterdam UMC, Location VUmc, Department of Epidemiology and Data Science, Amsterdam, Netherlands

**Keywords:** Knee osteoarthritis, Exercise therapy, Weight loss intervention, Qualitative study, Stratified care, Phenotypes

## Abstract

**Background:**

We have developed a model of stratified exercise therapy that distinguishes three knee osteoarthritis (OA) subgroups (‘high muscle strength subgroup’, ‘low muscle strength subgroup’, ‘obesity subgroup’), which are provided subgroup-specific exercise therapy (supplemented by a dietary intervention for the ‘obesity subgroup’). In a large clinical trial, this intervention was found to be no more effective than usual exercise therapy. The present qualitative study aimed to explore experiences from users of this intervention, in order to identify possible improvements.

**Methods:**

Qualitative research design embedded within a cluster randomized controlled trial in a primary care setting. A random sample from the experimental arm (i.e., 15 patients, 11 physiotherapists and 5 dieticians) was interviewed on their experiences with receiving or applying the intervention. Qualitative data from these semi-structured interviews were thematically analysed.

**Results:**

We identified four themes: one theme regarding the positive experiences with the intervention and three themes regarding perceived barriers. Although users from all 3 perspectives (patients, physiotherapists and dieticians) generally perceived the intervention as having added value, we also identified several barriers, especially for the ‘obesity subgroup’. In this ‘obesity subgroup’, physiotherapists perceived obesity as difficult to address, dieticians reported that more consultations are needed to reach sustainable weight loss and both physiotherapists and dieticians reported a lack of interprofessional collaboration. In the ‘high muscle strength subgroup’, the low number of supervised sessions was perceived as a barrier by some patients and physiotherapists, but as a facilitator by others. A final theme addressed barriers to knee OA treatment in general, with lack of motivation as the most prominent of these.

**Conclusion:**

Our qualitative study revealed a number of barriers to effective application of the stratified exercise therapy, especially for the ‘obesity subgroup’. Based on these barriers, the intervention and its implementation could possibly be improved. Moreover, these barriers are likely to account at least partly for the lack of superiority over usual exercise therapy.

**Trial registration:**

The Netherlands National Trial Register (NTR): NL7463 (date of registration: 8 January 2019).

**Supplementary Information:**

The online version contains supplementary material available at 10.1186/s12891-022-05496-2.

## Introduction

Knee osteoarthritis (OA) is a chronic joint disease that is characterized by large heterogeneity in aetiology, onset, course and treatment response among patients [1]. To better understand the disease and its treatment, the knee OA population may need to be classified into homogeneous phenotypes or subgroups of patients [1, 2]. By identifying clinically relevant subgroups, better tailored treatments could be offered, thereby optimizing clinical and economic outcomes [3].

**Fig. 1:**
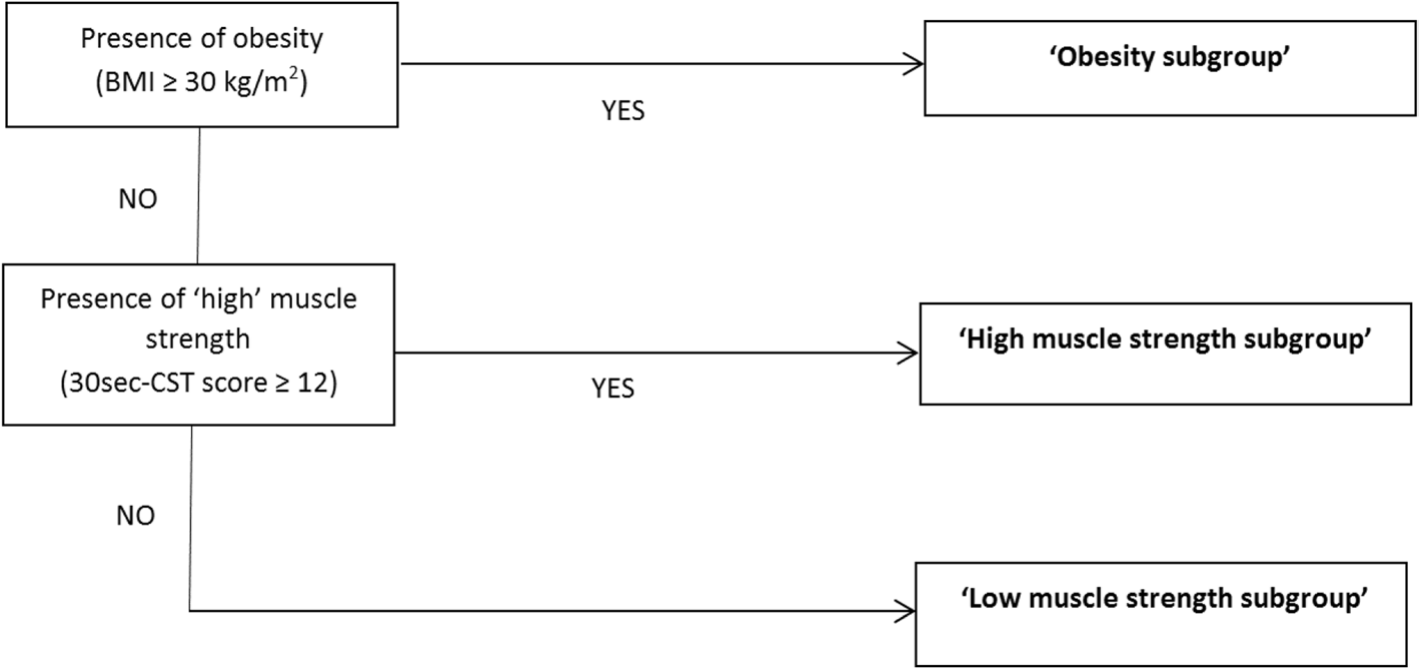
OCTOPuS stratification algorithm. BMI = body mass index, 30 s-CST = 30 s chair stand test, as measure for upper leg muscle strength

In previous work [4, 5], we identified potentially relevant and easy distinguishable subgroups from a large knee OA cohort, which we named the ‘high muscle strength subgroup’, ‘low muscle strength subgroup’ and ‘obesity subgroup’. Based on these three subgroups, we developed a model of stratified exercise therapy, consisting of (i) a stratification algorithm (see Fig. 1) that allocates patients into a subgroup, and (ii) a protocol for physiotherapists to deliver subgroup-specific exercise therapy, supplemented by a weight loss intervention from a dietician for the ‘obesity subgroup’ (see Table 1 for summary and Knoop et al [6]). This model was expected to result in larger effects on physical function and knee pain with lower costs, compared to the modest effects of usual exercise therapy [7]. A feasibility study [8] supported this expectation. However, in contrast to our hypothesis, stratified exercise therapy was not found to be more (cost-)effective than usual exercise therapy in a cluster randomized controlled trial (OCTOPuS-trial), in neither of the three subgroups [9, 10].

**Table 1:** Description of subgroup-specific, protocolized interventions

‘High muscle strength subgroup’	‘Low muscle strength subgroup’	‘Obesity subgroup’
Exercise therapy from physiotherapist (3–4 sessions + 1 ‘booster’ session): - subgroup-specific education/advice - home exercises	Exercise therapy from physiotherapist (8–12 sessions + 1–2 ‘booster’ sessions): - subgroup-specific education/advice - muscle strength training - home exercises	Exercise therapy from physiotherapist (12–18 sessions + 2–3 ‘booster’ sessions): - subgroup-specific education/advice - (adapted) muscle strength/aerobic training - home exercises
		Weight loss intervention from dietician (5–8 sessions): - advising and monitoring of healthy diet and active lifestyle
		Interprofessional consultation between physiotherapist and dietician

Alongside the OCTOPUS-trial, we performed a qualitative study to explore experiences from users (i.e., patients, physiotherapists and dieticians) with the intervention.. These qualitative data were expected to yield useful information to identify possible improvements in the intervention and its implementation. In addition, once we found that the intervention showed no superior effects over usual exercise therapy [9, 10], we also used these qualitative data to better understand the underlying reasons for not finding an effect of the intervention compared to usual exercise therapy.

The aim of this study is therefore to explore the experiences with stratified exercise therapy from the patient’s, physiotherapist’s and dietician’s perspective.

## Material and methods

### Study design

To explore users’ experiences with stratified exercise therapy in primary care, a qualitative study was carried out [11]. With this qualitative approach, we aimed to gain in-depth understanding of possibilities for improving the intervention and its implementation. In addition, once we found that the intervention showed no superior effects over usual exercise therapy [9, 10], we also used these qualitative data to better understand this finding. We followed the Standards for Reporting Qualitative Research [12] (SRQR) (see Supplementary file 1 for checklist).

The study was conducted in accordance with the Declaration of Helsinki and the ethical guidelines of the Vrije Universiteit Amsterdam**.** Ethical approval was obtained from the Medical Ethical Committee of the VU University Medical Centre (2018.563). All participants gave informed written consent. Confidentiality was maintained using restricted, secure access to the data, destruction of audio tapes following transcription and anonymizing of transcripts.

### Participant selection and recruitment

Patient inclusion into the OCTOPuS-trial was between January 2019 and May 2020. Full description of inclusion and exclusion criteria are described elsewhere [6]. In summary, patients were eligible if having had knee pain for at least three months and meeting the criteria for the clinical diagnosis of knee OA from the American College of Rheumatology [13].

Prior to the qualitative evaluation, we expected the number of interviews necessary for data saturation to be five patients in each subgroup (15 patients in total), ten physiotherapists and five dieticians, all from the experimental arm. Physiotherapists and dieticians could participate in the qualitative evaluation if they had treated at least three or two patients, respectively. For all eligible candidates, we used a random number generator to randomly select patients, physiotherapists and dieticians (i.e., random sampling). When a candidate refused to participate, the next eligible candidate from the random number generator was asked.

### Data collection

Semi-structured interviews were conducted between March and May 2020 using an interview guide (Supplementary file 2). An interview guide was developed with topics and questions derived from multiple sources (i.e., available knowledge on experiences with exercise therapy and diet in general, insights from our feasibility study [8], and experiences (advantages and disadvantages) of users with each of the components of the experimental intervention). The questions were open ended, to give the respondents the opportunity to bring up own topics that were not present in the interview guide. After each interview the interview guide was adapted when considered necessary. Since data collection took place during the COVID-19 pandemic, participants were interviewed by telephone. Interviews were performed by a female junior researcher (JWdJ) with five years of experience in qualitative research, who was not a clinician, had no specific knowledge of knee OA and physiotherapy and was not involved in the development of the model. Eligible participants were approached by e-mail or phone and asked to participate. If willing to participate, they provided informed consent. The interviews with patients and dieticians were approximately 15–30 min; with physiotherapists approximately 30–60 min. Interviews were audio-recorded, transcribed verbatim and anonymised with each participant given a coded number. To increase the credibility of the study, participants were sent a summary of their interview to check if they agreed with this summary (i.e., whether this summary adequately reflects their answers and opinions). Most participants (84%) agreed with the summary, while 10% requested small adaptations (i.e., mostly an addition of relevant information to the summary) and 6% did not respond.

### Data analysis

Data were analysed using elements of the methodology of the Grounded Theory Approach, namely the method of open, axial and selective coding [11]. First, open coding was applied by providing a label to each quote that could be related to a specific experience, for each of the three perspectives (i.e., patients, physiotherapists, dieticians), by two researchers (JK (PhD) and JWdJ (MSc)), independently from each other. Second, axial coding was applied by comparing all labels between the two researchers to reach agreement and to redefine if needed and then clustering them into themes and subthemes. Third, selective coding was applied by formulating overarching themes. To ensure confirmability, themes and subthemes were discussed with a third researcher (HB (PhD)) highly experienced in qualitative research. Software program MAXQDA version 2020 was used to facilitate data analysis.

In addition, we described general characteristics of the interviewed patients (i.e., age, gender, severity of symptoms and comorbidities) and of the physiotherapists and dieticians (i.e., age, gender, amount of work experience and number of patients treated in the OCTOPuS-trial).

## Results

**Fig. 2:**
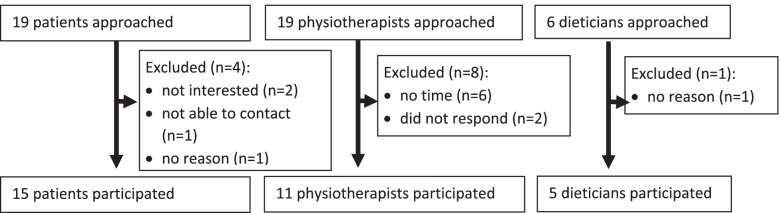
Flow chart of qualitative study

Interviews were conducted with a random sample from the experimental arm of 15 patients (five from each subgroup; from a total of 153 patients), eleven physiotherapists (from a total of 21 eligible physiotherapists) and five dieticians (from a total of six eligible dieticians) (see Fig. 2). Eleven instead of the intended ten physiotherapists were included in order to reach data saturation for the ‘obesity subgroup’. Characteristics of the respondents are described in Table 2 (for patients) and Table 3 (for physiotherapists and dieticians).

**Table 2:** Patient characteristics

Nr	Age	Sex	Pain severity (NRS 0–10)	Duration of symptoms (years)	Nr. sessions received	Other comments
P1-HMS	60	F	5	12	4 (PT)	
P2-HMS	52	F	6	25	3 (PT)	Severe comorbidity (cancer)
P3-HMS	54	F	3	3	7 (PT)	
P4-HMS	72	F	7	20	1 (PT)	Withdrawn from PT treatment (reason: costs)
P5-HMS	78	M	4	15	6 (PT)	
P6-LMS	74	F	3	10	12 (PT)	
P7-LMS	72	F	7	6	8 (PT)	
P8-LMS	49	F	6	36	12 (PT)	Severe comorbidity (back pain)
P9-LMS	68	M	8	2	8 (PT)	
P10-LMS	74	F	3	1	5 (PT)	Severe comorbidity (back pain)
P11-OS	58	F	4	1	10 (PT); 2 (D)	
P12-OS	65	F	8	35	9 (PT); 4 (D)	
P13-OS	46	F	8	20	12 (PT); 3 (D)	Withdrawn from diet treatment (reason: follows diet herself)
P14-OS	62	F	3	3	11 (PT); 1 (D)	Did already follow diet unsupervised
P15-OS	45	F	3	1	24 (FT); 2 (D)	Did already consulted dietician prior to start study

*M* Male, *F* Female, *HMS* ‘High muscle strength subgroup’, *LMS* ‘Low muscle strength subgroup’, *OS* ‘Obesity subgroup’, *PT* Physiotherapist, *D* Dietician

**Table 3:** Physiotherapist and dietician characteristics

**Nr**	**Age**	**Sex**	**Experience (in years)**	**Nr of participants**^a^
**Physiotherapists**				
PT1	26	F	5	5 (2–2-1)
PT2	30	M	5	3 (1–0-2)
PT3	42	M	20	3 (0–1-2)
PT4	30	M	5	6 (3–1-2)
PT5	39	F	17	5 (2–3-0)
PT6	48	F	24	5 (1–4-0)
PT7	65	M	39	5 (4–1-0)
PT8	24	M	1	3 (4–0-0)
PT9	26	F	2	5 (5–0-0)
PT10	30	M	7	4 (1–3-0)
PT11	58	F	33	4 (3–0-1)
**Dieticians**				
D1	35	F	4.5	8
D2	30	F	8	2
D3	47	F	23	3
D4	66	F	45	3
D5	54	F	32	2

*M* Male, *F* Female

^a^between brackets: number of patients in ‘high muscle strength subgroup’, ‘low muscle strength subgroup’ and ‘obesity subgroup’

In total, we identified four themes (with multiple subthemes), of which one theme addressed the positive experiences of the model and three themes the barriers. All (sub)themes and illustrative quotes will be described below, with Table 4 providing a concise overview of the (sub)themes and Table 5 providing an extended overview of (sub)themes and illustrative quotes.

**Table 4:** Overview of (sub)themes

Theme	Subtheme
1. Perceived added value of model	1a. Stratified approach highly appreciated but needs some flexibility
	1b. Exercises: effective and easy-to-perform
	1c. Patient education: important for coping and self-management skills
	1d. Booster sessions: contrary beliefs regarding their necessity
2. Difficulties in realizing the potential of combined treatment of ‘obesity subgroup’	3a. Obesity is a difficult topic to address
	3b. More is needed to reach sustainable weight loss
	3c. Poor collaboration between physiotherapist and dietician
3. Mixed feelings on minimal supervision of ‘high muscle strength subgroup’	
4. Barriers to knee OA treatment in general	4a. Lack of motivation
	4b. Comorbidity
	4c. Costs
	4d. Personal factors
	4e. COVID-19 lock-down

**Table 5:** Description of identified themes, subthemes and examples of illustrative quotes

Theme	Subtheme	Examples of illustrative quotes
***1. Perceived added value of model***	***1a. Stratified approach appreciated but only needs some flexibility***	*“This time the physiotherapist really listened to me and gave me exercises tailored to my situation. While usually, a physiotherapist gives you a squat exercise, while you cannot do this exercise. But now, the physiotherapist did some subtle changes to the exercises, so I could really perform them and keep performing them.”* (P13-OS)
		“*Usually, we provided one standard treatment program to everybody. I suppose that this was too heavy for some and too little for others. So I think it is really smart to distinguish subgroups and to differentiate the treatment to each subgroup*.” (PT17)
		*“This differentiation [of people with low versus high muscle strength] was something we always did.”* (PT6)
		*“When you see a patient for the first time, you see quite easily in which subgroup he or she will be allocated, which was confirmed by the stratification algorithm.”* (PT10)
		*“Perhaps, a patient who only just reaches the cut-off of 12 repetitions [at 30-s chair stand test] should possibly be allocated to the low muscle strength subgroup.”* (PT5)
	***1b. Exercises: effective and easy-to-perform***	*“Most of the exercises were very easy to perform, so I could do it at home as well.”*(P7-LMS)
		*“I see a clear difference with the exercises from my previous physiotherapist. Those were a lot lighter and not really to strengthen my muscles but to make my muscles more flexible. But the physiotherapist from the OCTOPuS-trial really focused at strengthening of the upper leg muscles.”* (P11-OS)
		*“What helped me [in persisting to perform home exercises] is that when I did not exercise for a day, my knee became more painful, so you think: “that’s your own fault, you should have done your exercises.”*(P6-LMS)
	***1c. Patient education: important for coping and self-management skills***	*"It was a shock when I heard that I have OA, because I was young and believed that I should be very cautious. But now it was explained that this is not the case, and that I can still do almost anything.”* (P2-HMS)
	***1d. Booster sessions: contrary beliefs regarding their necessity***	“*It varies whether a booster sessions is helpful. Some of my patients had almost no symptoms anymore at the end the treatment, so I hesitated to suggest a booster session. But some others still had significant symptoms at the end, who I wanted to come back after a while.”* (PT9) *“The physiotherapist had no control at all whether I did my home exercises or not. While when you visit the physiotherapist every week, you are inclined to keep performing the exercises every week.”* (P11-OS)
***2. Difficulties in realizing the potential of combined treatment in ‘obesity subgroup’***	***2a. Obesity is a difficult topic to address***	*“The name of the subgroup was tricky, because then you directly address obesity. Patients mostly do not appreciate this.”* (PT4)
		*“There is quite a threshold for me to discuss obesity, but as the treatment protocol recommends to address this, I just did this and I felt less burdened.”* (PT1)
	***2b. More is needed to reach sustainable weight loss***	*“When you lose weight, you benefit from two sides: you are feeling fitter and can perform your exercises more easily, so that is really positive.”* (P14-OS)
		*“It makes the treatment more professional, for example in your communication to the patient, as you can say:* “I have discussed this with your dietician…” *It gives you more confidence in your treatment, and I assume that it gives the patient more confidence in the treatment as well. Because there are two professionals working together and knowing more than one.”* (PT1)
		*“For me this is a very good combination. As the knee complaints can be reduced by the physiotherapists as well as by losing weight.”* (D2)
		*“I think that when you really strive for long-lasting weight loss, than the 3 h [maximal time than can be reimbursed] is not sufficient. While especially when you combine your treatment with a physiotherapy treatment for at least 1 year, than you can expect some real change.” (D4)*
		*“Because I already started with a fitness-coach, I did not see the need for consulting a dietician as well.”* (P11-OS)
	***2c. Poor collaboration between physiotherapist and dietician***	*“I have to confess that the collaboration did not go very easy so far.”* (PT10)
		*“It is really nice that she [the dietician] is located inside our practice, so we can easily and shortly consult each other on our shared patients.”* (PT3)
		*“This [collaboration with physiotherapist] was a difficult component, which was mostly due to the location where I see those patients. As I am only located at the physiotherapy practice once every 2 weeks, I do not see the physiotherapists on a regular base. Mostly, we only discussed those patients that did not go very well, but it was difficult to initiate a more structural consultation.”* (D1)
***3. Mixed feelings on minimal supervision of ‘high muscle strength subgroup’***		“*Sometimes, a patient had to train at home for 6 weeks without any supervision, so as a physiotherapist you lack control in preventing joint overloading. I can only try to advice the patient where they should pay attention to and how to recognize overloading, but you cannot check this.”*(PT1)
		*”I would have preferred some more contact, as this would have motivated me to continue.”* (P1-HMS)
		*“They were mostly relatively young male people doing sports. So yes, those people do not have to train under supervision.”* (PT5)
		*“I think this is a better way, because now the responsibility is fully yours and you know what you should do. I like that”* (P3-HMS)
***4. Barriers for knee OA treatment in general***	***4a. Lack of motivation***	*“Personally, I find it very hard to keep being motivated, and to make time to do your exercises.”* (P11-OS)
	***4b. Comorbidity***	*“One patient had balance problems, therefore I could not let her perform the exercises on her own. That’s why I could not strictly follow the treatment protocol.”* (PT1)
	***4c. Costs***	*“Because of the start of a new year, I have to pay my own risk first. For me this is too much money in comparison to the small benefits [of the diet intervention].”* (P15-OS)
		*“I did not exceed the recommended 3 h in my patients, but I think that more is necessary. That is widely known. However, these extra hours will not be reimbursed and people are not willing to pay for this.”* (D3)
	***4d. Personal factors***	*“Because something happened in his family, he wanted the treatment to be on hold for a while.”* (D5)
	***4e. COVID-19 lock-down***	*“Only just after overcoming my barriers and visiting the gym 2 times a week, the corona-crisis started and the gym closed.”* (P15-OS)

### Theme 1: Perceived added value of model

#### Subtheme 1a. Stratified approach highly appreciated but needs more flexibility

All physiotherapists stated that they found the stratified approach facilitative for tailoring of the treatment. Also patients experienced a more tailored approach, in which their treatment was truly personalized to their needs, compared to previous treatments that they had received from physiotherapists (as illustrated by this quote):

Patient #13 (‘obesity subgroup’): *“This time the physiotherapist really listened to me and gave me exercises tailored to my situation. While usually, a physiotherapist gives you a squat exercise, while you cannot do this exercise. But now, the physiotherapist did some subtle changes to the exercises, so I could really perform them and keep performing them.”*

Most physiotherapists intended to continue providing this stratified approach after completion of the OCTOPuS-trial. For some physiotherapists, the stratified approach was new, while others reported that they already used such an approach, as illustrated by the following contrasting quotes:

Physiotherapist #17: “*Usually, we provided one standard treatment program to everybody. I suppose that this was too heavy for some and too little for others. So I think it is really smart to distinguish subgroups and to differentiate the treatment to each subgroup*.”

Physiotherapist #6: *“This differentiation [of people with low versus high muscle strength] was something we always did.”*

Nearly all physiotherapists agreed with the identification of the three subgroups in the model, but a small number felt the need for a fourth subgroup (e.g., an intermediate subgroup between the ‘high muscle strength subgroup’ and’low muscle strength subgroup’, or a subgroup of elderly people with comorbidities). Physiotherapists were unanimously positive about the method of allocating patients into subgroups (i.e., tests, cut-off values and algorithm). They found this method easy and quick to apply. Moreover, most physiotherapists agreed with the cut-off values in the stratification algorithm, with subgroup allocations generally in line with their own expectations of appropriate subgroups for their patients. Finally, the physiotherapists were generally positive about the training that they received prior to the study (i.e., one training course, supplemented by online video’s and site visits by researcher team member). Physiotherapists unanimously stated that at the start of the trial, they felt capable of adequately applying the intervention.

The only disadvantage mentioned by some physiotherapists was the lack of flexibility in the algorithm to deviate from it, for patients with scores close to the cut-offs, as illustrated by this quote:

Physiotherapist #5: *“Perhaps, a patient who only just reaches the cut-off of 12 repetitions [at 30-seconds chair stand test] should possibly be allocated to the low muscle strength subgroup.”*

#### Subtheme 1b. Exercises: effective and easy-to-perform

Physiotherapists and patients were generally positive about the (predominantly strengthening) exercises in each of the three subgroup-specific treatment protocols. The simplicity of the exercises was especially appreciated, as this enabled patients to perform the exercises at home after only a small number of supervised sessions, as illustrated by the following quote:

Patient #7 (‘low muscle strength subgroup’): *“Most of the exercises were very easy to perform, so I could do it at home as well.”*

Patients were also positive about the intensity of the muscle strengthening exercises, being mostly higher than in previous physiotherapy treatments. In addition, most patients reported having experienced pain relief after exercising for some weeks and some observed an increase in knee symptoms when they had not exercised for a few days. Both aspects were found to facilitate their motivation to keep exercise.

#### Subtheme 1c. Patient education: important for coping and self-management

Many patients reported that they benefitted from the information as provided by the physiotherapist on how to manage their knee OA symptoms. Following physiotherapists’ explanation, they realized that physical activity would not damage their knee joints. This helped them to stay motivated to perform the exercises and increase the intensity of their exercise programmes, as illustrated by this quote:

Patient #2 (‘high muscle strength subgroup’): *"It was a shock when I heard that I have OA, because I was young and believed that I should be very cautious. But now it was explained that this is not the case, and that I can still do almost anything.”*

#### Subtheme 1d. Booster sessions: contrary beliefs around their necessity

When discussing the role of so-called booster or refreshment sessions during the follow-up period, many patients and physiotherapists reported that no booster sessions had (yet) been provided. Some physiotherapists explained this by reporting that there was no need for a booster session, as the patient was able to exercise and manage their symptoms on their own. In many cases, it was agreed that the patient would contact the physiotherapist for a booster session if symptoms became exacerbated, but this did not usually occur. These contrary beliefs are nicely illustrated by the following quote:

Physiotherapist #9: “*It varies whether a booster sessions is helpful. Some of my patients had almost no symptoms anymore at the end the treatment, so I hesitated to suggest a booster session. But some others still had significant symptoms at the end, who I wanted to come back after a while.”*

On the other hand, some physiotherapists and patients noted a lack of monitoring after the treatment phase, resulting in a lack of motivation for the patients to keep exercising (for which the booster sessions had been recommended), as illustrated by this quote:

Patient #11 (‘obesity subgroup’): *“The physiotherapist had no control at all whether I did my home exercises or not. While when you visit the physiotherapist every week, you are inclined to keep performing the exercises every week.”*

### Theme 2: Difficulties in realizing the potential of combined treatment of ‘obesity subgroup’

#### Subtheme 2.1: Obesity is a difficult topic to address

Most physiotherapists reported feeling uncomfortable about discussing obesity and the need for weight loss with patients, fearing that raising this topic could jeopardize their relationship, or feeling that they lacked the skills to address this topic appropriately. This is illustrated by the following quote:

Physiotherapist #14: *The name of the subgroup was tricky, because then you directly address obesity. Patients mostly do not appreciate this.”*

On the other hand, the subgroup-specific approach encouraged them to attempt to address the topic.

#### Subtheme 2b: More is needed to reach sustainable weight loss

Generally, all patients, physiotherapists and dieticians agreed that it is essential to combine physiotherapy with a diet intervention in this subgroup, as illustrated by this quote:

Patient #14 (‘obesity subgroup’): “When you lose weight, you benefit from two sides: you are feeling fitter and can perform your exercises more easily, so that is really positive.”

Moreover, physiotherapists were positive about the extended number of recommended sessions for this subgroup, as this enabled them to supervise and control the performance of exercises more closely and to facilitate exercise adherence more effectively.

On the other hand, dieticians unanimously reported that their recommended number of sessions (i.e., 5 to 8 sessions, with a total duration of 3 h as this is the maximal time covered by health insurance) is definitely not enough for reaching sustainable weight loss, as illustrated by the following quote:

Dietician #4: *“I think that when you really strive for long-lasting weight loss, than the 3 hours [maximal time than can be reimbursed] is not sufficient. While especially when you combine your treatment with a physiotherapy treatment for at least 1 year, than you can expect some real change.”*

Moreover, when patients were already following a diet restriction by themselves or had negative experiences with previous diet restrictions, they were mostly unwilling to spend time for sessions with a dietician.

#### Subtheme 2c: Poor collaboration between physiotherapist and dietician

Both physiotherapists and dieticians reported difficulties with the recommended collaboration. In many cases, each waited for the other to initiate collaboration or only considered its possibility when difficulties arose in the treatment. It appeared that the location of the dietician was crucial for the interdisciplinary consultation. When dieticians and physiotherapists were located in the same building, they consulted each other easily, while being located in different locations was reported as a major barrier. This collaboration depending on the location is illustrated by the following quote:

Dietician #1: *“This [collaboration with physiotherapist] was a difficult component, which was mostly due to the location where I see those patients. As I am only located at the physiotherapy practice once every 2 weeks, I do not see the physiotherapists on a regular base. Mostly, we only discussed those patients that did not go very well, but it was difficult to initiate a more structural consultation.”*

### Theme 3: Mixed feelings on minimal supervision of ‘high muscle strength subgroup’

When addressing the ‘high muscle strength subgroup’ in the interviews, the low number of sessions was the most important topic. Many physiotherapists mentioned their struggle to adhere to this low number of sessions – being three to five—due to a perceived lack of control over the treatment. Moreover, some of them questioned whether they could effectively treat patients from this subgroup with such a low level of supervision. Such negative experiences were also reported by some patients from this subgroup, who preferred more sessions to enhance their discipline to keep exercising. The following two quotes are illustrative for these negative feelings regarding the minimal supervision.

Physiotherapist #1: “*Sometimes, a patient had to train at home for 6 weeks without any supervision, so as a physiotherapist you lack control in preventing joint overloading. I can only try to advice the patient where they should pay attention to and how to recognize overloading, but you cannot check this.”*

Patient #1 (‘high muscle strength subgroup’): *”I would have preferred some more contact, as this would have motivated me to continue.”*

On the other hand, many other physiotherapists reported that they viewed this minimal supervision positively, as it facilitated a ‘hands-off approach’ with a stronger focus on self-management. Several patients also preferred the minimal level of supervision, as they merely wanted specific advices to prevent knee overloading as already being able to exercise for themselves. The following two quotes illustrate these positive feelings:

Physiotherapist #5: *“They were mostly relatively young male people doing sports. So yes, those people do not have to train under supervision.”*

Patient #3 (‘high muscle strength subgroup’): *“I think this is a better way, because now the responsibility is fully yours and you know what you should do. I like that.”*

### Theme 4: Barriers to knee OA treatment in general

#### Subtheme 4a: ‘Lack of motivation’

From all three perspectives, a lack of motivation and discipline was reported as main barrier to optimal provision of the intervention (as illustrated by the following quote of a patient), while high motivation was reported as main facilitator of a successful treatment.

Patient #11 (‘obesity subgroup’): *“Personally, I find it very hard to keep being motivated, and to make time to do your exercises.”*

#### Subtheme 4b: ‘Comorbidities’

Both physiotherapists and dieticians mentioned patients whose other health issues negatively influenced their treatment (as illustrated by the quote provided below) or even resulted in their dropping out. Unexpectedly, this was most often reported for the’low muscle strength subgroup’, while comorbidities were more expected to affect the treatment were expected in the ‘obesity subgroup’.

Physiotherapist #1: *“One patient had balance problems, therefore I could not let her perform the exercises on her own. That’s why I could not strictly follow the treatment protocol.”*

#### Subtheme 4c: Costs

Most patients reported that their health insurance covered their treatments, but some mentioned that costs were a problem. This mainly concerned the ‘own contribution’ component (meaning for each calendar year, the first €385 of physiotherapy or dietary treatments is to be paid by the patient) or the allowed maximum of twelve physiotherapy sessions and three hours for dietary consults covered by the health insurance company, resulting in some patients not being willing to exceed this number, as illustrated by this quote:

Dietician #3: *“I did not exceed the recommended three hours in my patients, but I think that more is necessary. That is widely known. However, these extra hours will not be reimbursed and people are not willing to pay for this.”* (D3)

#### Subtheme 4d: Personal factors

In several cases, personal factors (e.g. mental stress and/or time constraints) were reported as barriers to the treatment. These factors meant that some patients did not adhere to the home exercises or diet advice, or cancelled their physiotherapy or dietician sessions, as illustrated by a dieticians’ quote:

Dietician #5: *“Because something happened in his family, he wanted the treatment to be on hold for a while.”*

#### Subtheme 4e: COVID-19 lock-down

Finally, the temporary closure of health care sites and fitness centers during the lock-down period in the Netherlands was considered an important barrier by several patients. Because of these closures, treatments were ended prematurely or given online and patients reported missing the routine of visiting a fitness centre for their (home) exercises, as illustrated by this quote from a patient:

Patient #15 (‘obesity subgroup’): *“Only just after overcoming my barriers and visiting the gym two times a week, the corona-crisis started and the gym closed.”*

## Discussion

This qualitative study explored the experiences of patients with knee OA, of physiotherapists and of dieticians with stratified exercise therapy, as applied in the OCTOPuS-trial. In general, the intervention was found to have added value in clinical practice, but a number of generic and subgroup-specific barriers were reported. These barriers are likely to account – at least partly—for the lack of superiority in clinical and economic effects of stratified exercise therapy, when compared to usual exercise therapy, as found in the OCTOPuS-trial [9, 10], and should be addressed in a revised model if possible.

Overall, positive experiences with the stratified approach of our model were reported from all three perspectives (i.e., patients, physiotherapists, dieticians), which on the one hand is in line with previous qualitative studies [4, 5, 8], but on the other hand is in contrast to the lack of (cost-)effectiveness found in our OCTOPuS-trial [9, 10] and recent other trials on stratified exercise approaches [14–18]. Physiotherapists generally intended to continue providing this stratified approach after completion of the OCTOPuS-trial and both dieticians and physiotherapists were enthusiastic about combining their professional expertise for the ‘obesity subgroup’ to achieve the best outcomes for these patients. Patients were pleasantly surprised by the personalized approach in their intervention, in which the content and type of delivery of the treatment was adapted to their specific subgroup. Many patients reported that their physiotherapist ‘*really listened’* and that this was in contrast to previous physiotherapy treatments. These findings emphasize that, although a personalized approach is already generally acknowledged as being crucial for effective physiotherapy treatments [19], this needs to be addressed more extensively in physiotherapy education [20]. The personalized approach, however, could also make our stratified approach redundant, if the treatment is not only tailored to the stratification factors of our model (i.e., upper leg muscle strength and BMI) but also to many other relevant factors. Moreover, this personalization is also a possible reason – next to the observed barriers—for the lack of effect of stratified compared, as it might already be applied in usual care and therefore minimizes the contrast with our intervention.

As well as facilitators, we also identified several barriers that should be addressed in a revised model if possible. One barrier was found for the model in general, namely the inflexibility of the stratification algorithm. Some physiotherapists would have preferred a degree of flexibility in applying the algorithm. This would enable them to incorporate their clinical expertise in this decision and, when considered necessary, deviate from the algorithm. Moreover, especially in the physiotherapy context, a stratification algorithm might be too simplistic, as a large number of patient factors and preferences should be taken into account to provide optimal, personalized care [21]. Therefore, we propose to allow flexibility in applying an algorithm (or other clinical decision support tool), so that clinical expertise maintains the key factor in the clinical decision making process, in line with Greenhalgh et al. [21]

We also collected contrary beliefs regarding the use of booster sessions, which perhaps reflects the conflicting evidence on their added value [22–25]. It appears that many primary care physiotherapists tended to end their treatment after a relatively short period (such as three months), and did not monitor their patients afterwards. It is likely that a small number of booster sessions after the treatment period would be helpful for sustaining exercise behavior changes, therefore maintaining the beneficial effects of exercise therapy over time. Future research is needed to clarify whether or not these booster sessions are effective or not.

Subgroup-specific barriers were especially found to the’obesity subgroup’, which appeared to be the most challenging subgroup. The first main barrier in the obesity subgroup is the difficulty for physiotherapists of discussing obesity with the patient, which has also been found in other studies [26–28]. Therefore, it is crucial – especially given the increasing prevalence of obesity in society [29] – that physiotherapists be educated more extensively in how to address obesity. Apparently, the training that was provided to experimental arm physiotherapists prior to the OCTOPuS-trial (consisting of a training course, online videos and site visits by the researcher) was not sufficient for this specific purpose.

The second main barrier in the ‘obesity subgroup’ was that dieticians perceived the level of supervision (i.e., maximum of 150 min in total) as insufficient to reach sustainable weight loss, especially in those patients that (unsuccessfully) had been or were following weight restrictions before. This could explain the failure of the weight loss intervention in the OCTOPuS-trial, as the ‘obesity subgroup’ on average did not lose any weight [9]. Remarkably, many dieticians provided even less sessions as recommended. Dieticians may need to extend the use of behavioral change techniques (e.g. by using e-health), to be more successful in this subgroup.

The third main barrier in the ‘obesity subgroup’ concerned the recommended consultation between physiotherapist and dietician. Although the benefits of such interprofessional collaboration were recognized by both sides, each reported major barriers to its implementation (which was found by others as well [30, 31]). A clear division of tasks, in which one is appointed as ‘case-manager’ and therefore initiator of interprofessional consultation, might help reduce this barrier. Because of their key position and expertise in knee OA management [32], we propose that physiotherapists take this case manager role.

With regards to the ‘high muscle strength subgroup’, the minimal supervision was found challenging by some physiotherapists and patients as it hindered an establishment of their relationship. This minimal supervision is substantially lower than usually applied in knee OA treatment in the Netherlands (i.e., 3–5 vs. 17 physiotherapy sessions [33]). Recent work showed that a so-called ‘therapeutic alliance’ could be a crucial condition for an effective treatment [20], which underlines the reported concerns. On the other hand, a number of studies has shown that minimally supervised exercise therapy interventions (e.g., web-based [34, 35], Skype-based [36], telephone-based [37] or a combination of web-based and face-to-face interventions [38, 39]) are not necessarily inferior to fully supervised interventions and may even be superior in the longer term, due to their focus on self-management. Our OCTOPuS-trial confirms this conclusion, as stratified exercise therapy resulted in similar effects as usual exercise therapy in the ‘high muscle strength subgroup’, while saving a number of physiotherapy sessions in the meantime [9]. Future research should therefore clarify for which patients the physiotherapy treatment can be provided more efficient (that is, same clinical effect in less sessions).

The barriers identified in this qualitative study are likely to account—at least partly for the lack of superiority of our intervention over usual exercise therapy [9, 10]. Moreover, these barriers imply that the intervention and its implementation could possibly be improved by more extended training of physiotherapists in (i) adequately addressing the topic ‘obesity’ and (ii) effectively collaborating with a dietician in the ‘obesity subgroup’, (iii) adequate guiding in only a minimal number of sessions of the ‘high muscle strength subgroup’, and (iv) adequate usage of booster sessions in all patients. Moreover, the model could possibly result in better outcomes when (i) more dietician sessions could be offered and (ii) more tools and behavioral change techniques could be applied to motivate patients for a longer term in performing exercises and/or maintain their healthy diet. Finally, we propose that some flexibility in applying the stratification algorithm should be allowed, similar to that allowed in the application of clinical guideline.

Two limitations of our study should be addressed. First, we interviewed a relatively small sample (15 patients, 11 physiotherapists, 5 dieticians), who were all motivated to participate in this additional qualitative study, and therefore possibly not representative of the total study population. Second, due to the COVID-19 restrictions, all interviews were conducted by phone, so that non-verbal signs could not be detected.

## Conclusions

This qualitative study has revealed that patients, physiotherapists and dieticians generally recognized the added value of our new model of stratified exercise therapy. However, a number of barriers to effective application of the intervention were also identified, especially in the ‘obesity subgroup’ (e.g., difficulty to address obesity by physiotherapists, too few dietary sessions, poor interprofessional collaboration physiotherapist/dietician). Based on these barriers, a number of improvements of the intervention and its implementation have been suggested. Moreover, these barriers are likely to account at least partly for the lack of superiority of this model over usual exercise therapy in the OCTOPuS-trial.

## Supplementary Information

**Additional file 1.** 

**Additional file 2.** 

## Data Availability

The research protocol of the trial is accessible at the Netherlands National Trial Register (https://www.trialregister.nl/trial/7463) and Open Science Framework (https://osf.io/x3p94/). The datasets used and/or analysed during the current study available from the corresponding author on reasonable request.

## Abbreviations

30 s-CST: 30 Seconds Chair Stand Test
BMI: Body Mass Index
NRS: Numeric Rating Scale
OA: OsteoArthritis
RCT: Randomized Controlled Trial
SRQR: Standards for Reporting Qualitative Research
