# Supplementary material for: Patients’ and clinicians’ experiences with stratified exercise therapy in knee osteoarthritis: a qualitative study
Source: BMC Musculoskelet Disord. 2022 Jun 9;23:559. doi: 10.1186/s12891-022-05496-2 (PMC9178540; doi:10.1186/s12891-022-05496-2)
Supplement: Supplementary file 1 — Additional file 1. [file 12891_2022_5496_MOESM1_ESM.docx]

**Supplementary file 1: Checklist of SRQR**

| **No.** | **Topic** | **Page** |
| --- | --- | --- |
|  | **Title and abstract** |  |
| **S1** | Title | 1 |
| **S2** | Abstract | 2 |
|  | **Introduction** |  |
| **S3** | Problem formulation | 4-5 |
| **S4** | Purpose or research question | 5 |
|  | **Methods** |  |
| **S5** | Qualitative approach and research paradigm | 6-7 |
| **S6** | Researcher characteristics and reflexivity | 6-7 |
| **S7** | Context | 6 |
| **S8** | Sampling strategy | 6 |
| **S9** | Ethical issues pertaining to human subjects | 6 |
| **S10** | Data collection methods | 6-8 |
| **S11** | Data collection instruments and technologies | 6-7 |
| **S12** | Units of study | 6-7 |
| **S13** | Data processing | 7 |
| **S14** | Data analysis | 7 |
| **S15** | Techniques to enhance trustworthiness | 7 |
|  | **Results/findings** |  |
| **S16** | Synthesis and interpretation | 9-15 |
| **S17** | Links to empirical data | 9-15 |
|  | **Discussion** |  |
| **S18** | Integration with previous work, implications, transferability, and contribution(s) to the field | 16-18 |
| **S19** | Limitations | 18-19 |
|  | **Other** |  |
| **S20** | Conflicts of interest | 22 |
| **S21** | Funding | 22-23 |
