# Supplementary material for: Patients’ and clinicians’ experiences with stratified exercise therapy in knee osteoarthritis: a qualitative study
Source: BMC Musculoskelet Disord. 2022 Jun 9;23:559. doi: 10.1186/s12891-022-05496-2 (PMC9178540; doi:10.1186/s12891-022-05496-2)
Supplement: Supplementary file 2 — Additional file 2. [file 12891_2022_5496_MOESM2_ESM.docx]

**Supplementary file 2**: **Interview guides**

**Interview guide physiotherapists**

1. **Educational session**
2. The education consisted of four components: (1) e-learning on new physiotherapy guideline for knee/hip OA, (2) session/workshop on intervention, (3) online videos of session/workshop (4) manual of intervention. What is your opinion of this education?
3. How many times did you watch the online videos?
4. Did you felt prepared to apply the intervention after following this education, or did you prefer additional education?
5. Do you (other) have suggestions for improvement of the education?
6. **In- and exclusion criteria**
7. What is your opinion of these criteria for patients to be eligible for our study?
8. What should we have done differently?
9. **Allocation into subgroups**
10. You allocated patients into subgroups based on two tests: BMI en 30-seconds chair stand test for upper leg muscle strength. What is your opinion of these two tests?
11. What is your opinion of the cut-off values in our stratification algorithm?
12. Do you feel this is the adequate method for allocating patients into subgroups?
13. What is your opinion of the three subgroups (high muscle strength subgroup, low muscle strength subgroup and obesity subgroup)? Are these the right subgroups?
14. What should we have done differently?
15. **Treatment (only regarding those subgroups that the physiotherapist have applied)**

***High muscle strength subgroup***

1. What is your opinion on the subgroup-specific treatment for this subgroup?
2. The focus was on prevention of knee overloading en instruction of home exercises. What is your opinion of this focus?
3. What is your opinion on the recommended number of sessions?
4. Did you provide ‘booster sessions’, and if so, how did you perceive its added value? If not, why not?
5. What was the distribution of time spend on education vs. exercise therapy in this subgroup?
6. What is your opinion on the subgroup-specific patient education material from the protocol? Do you use this, or did you use own education material?
7. What is your opinion on the (home) exercises recommended in the protocol? Did you also use other (home) exercises?
8. Did you achieve the maximal outcome in this subgroup? If not, what are the reasons?
9. Do you have (other) suggestions for improvement of the treatment in this subgroup?

***Low muscle strength subgroup***

1. What is your opinion on the subgroup-specific treatment for this subgroup?
2. The focus was on high intensity muscle strengthening exercises. What is your opinion of this focus?
3. What is your opinion on the recommended number of sessions?
4. Did you provide ‘booster sessions’, and if so, how did you perceive its added value? If not, why not?
5. What was the distribution of time spend on education vs. exercise therapy in this subgroup?
6. What is your opinion on the subgroup-specific patient education material from the protocol? Do you use this, or did you use own education material?
7. What is your opinion on the (home) exercises recommended in the protocol? Did you also use other (home) exercises?
8. Did you achieve the maximal outcome in this subgroup? If not, what are the reasons?
9. Do you have (other) suggestions for improvement of the treatment in this subgroup?

***Obesity subgroup***

1. What is your opinion on the subgroup-specific treatment for this subgroup?
2. The focus was on combining aerobic and muscle strengthening exercises, with a gradual increase in intensity. What is your opinion of this focus?
3. What is your opinion on the recommended number of sessions?
4. Did you provide ‘booster sessions’, and if so, how did you perceive its added value? If not, why not?
5. What was the distribution of time spend on education vs. exercise therapy in this subgroup?
6. What is your opinion on the subgroup-specific patient education material from the protocol? Do you use this, or did you use own education material?
7. What is your opinion on the (home) exercises recommended in the protocol? Did you also use other (home) exercises?
8. Did you apply the recommended adaptations of the exercises, to prevent knee pain exacerbation? If so, which one.
9. What is your opinion on the addition of a diet intervention? Does this has added value?
10. How did you perceive addressing obesity and the diet intervention at the patient?
11. How did you perceive the collaboration with the dietician?
12. Did you achieve the maximal outcome in this subgroup? If not, what are the reasons?
13. Do you have (other) suggestions for improvement of the treatment in this subgroup?
14. **General questions**
15. Which facilitators of a successful treatment did you perceive?
16. Which barriers of a successful treatment did you perceive?
17. Was the treatment different than you usually provide?
18. Will you continue providing this treatment after completion of the study? Why?
19. In case of nationwide implementation of this treatment, what should we bear in mind, and what should we change?
20. Do you have any further comments?

**Interview guide dieticians**

1. One subgroup (obesity subgroup) received a combined intervention from physiotherapist and dietician. What is your opinion on this combined treatment?
2. How did you perceive providing the diet intervention tot his subgroup?
3. What is your opinion on the recommended number of sessions? What this sufficient to achieve the goal of 10% weight loss in 1 year?
4. The treatment should consist of diet advice and physical activity advise. What kind of advices did you provide?
5. How did your patients perceive these advices?
6. What was the distribution of time spend on diet vs physical activity advice?
7. How did you perceive the collaboration with the physiotherapist? Did this perceive added value of this collaboration?
8. Did you already collaborate with a physiotherapist prior to our study?
9. Did you treat patients with knee OA before?
10. Which facilitators of a successful treatment did you perceive?
11. Which barriers of a successful treatment did you perceive?

**Interview guide patients**

The treatment in this OCTOPuS-study was not the same for everyone, but differed between certain subgroups. The treatment you received depended on the subgroup that you were allocated, based on your characteristics. Thereby, we aimed at tailoring the treatment to your situation.

1. Did you perceive your treatment as a tailored treatment? Why (not)?
2. What were the best elements of the treatment? Why?
3. What are elements of the treatment that should be improved? Why?
4. How was your relationship with the physiotherapist?
5. How did you judge the expertise of the physiotherapist?
6. Have you received physiotherapy treatment for your knee symptoms previously? If so, did you notice any differences between that previous treatment and your treatment in our study?

*The physiotherapy treatment consisted of multiple components: education/advices, supervised exercise therapy, and instruction of home exercises and physical activities.*

1. How did you perceive the education/advices from the physiotherapist? Which topics did you discuss?
2. How did you perceive the supervised exercise therapy?
3. How many sessions did you receive?
4. How did you perceive the instruction of home exercises and physical activities?
5. How many days per week did you perform these at home, during the treatment period, and during the period following this treatment period? Which home exercises and physical activities did you perform?
6. If performed frequently, what helped you in keep performing them frequently?
7. If not performed frequently, what would have helped you performing and/or keep performing these home exercises and physical activities more often?

***Only regarding the subgroup that the patient was allocated to:***

***High muscle strength subgroup***

*You were allocated to the high muscle strength subgroup. Your treatment from the physiotherapist consisted of only a few sessions, in which you primarily received advices on how to prevent knee overloading and instructions for home exercises.*

1. What is your opinion of this subgroup that you were allocated to? Did you agree with this?
2. What is your opinion of the number of sessions from the physiotherapist (too few, too much, exactly the right number)?
3. What is your opinion of the advices from the physiotherapist?
4. When the treatment ended, was it ended because the treatment goals had been achieved or for other reasons? (if other reason, which one?)

***Low muscle strength subgroup***

*You were allocated to the low muscle strength subgroup. Your treatment from the physiotherapist consisted of on average 12 sessions in which you primarily received muscle strengthening exercises.*

1. What is your opinion of this subgroup that you were allocated to? Did you agree with this?
2. What is your opinion of the number of sessions from the physiotherapist (too few, too much, exactly the right number)?
3. What is your opinion of the exercises from the physiotherapist?
4. When the treatment ended, was it ended because the treatment goals had been achieved or for other reasons? (if other reason, which one?)

***Obesity subgroup***

*You were allocated to the obesity subgroup. Your treatment from the physiotherapist consisted of on average 18 sessions in which you primarily received aerobic and muscle strengthening exercises, which were only slowly gradually increased in intensity to avoid pain exacerbation.*

1. What is your opinion of this subgroup that you were allocated to? Did you agree with this?
2. What is your opinion of the number of sessions from the physiotherapist (too few, too much, exactly the right number)?
3. What is your opinion of the slow gradual increase in intensity?
4. What is your opinion of the exercises from the physiotherapist?
5. When the treatment ended, was it ended because the treatment goals had been achieved or for other reasons? (if other reason, which one?)

*Next to the treatment from the physiotherapist, you also received a weight loss intervention from a dietician.*

1. What is your opinion of this additional treatment?
2. What is your opinion of the diet advices from the dietician?
3. What is your opinion of the physical activity advices from the dietician?
4. How was your relationship with the dietician?
5. How did you judge the expertise from the dietician?
6. Have you received a treatment from a dietician previously? If so, did you notice any differences between that previous treatment and your treatment in our study?
